# Radioprotective Effect of Ascorbic Acid: A Need for Prophylactic Supplementation Pre‐ and Post‐Radiation Exposure Among Patients and Healthcare Workers—A Perspective Article

**DOI:** 10.1002/hsr2.71996

**Published:** 2026-03-19

**Authors:** Justice Mushemba, Cornel M. Angolile, Jaynes F. Kabuhaya, Harold L. Mashauri

**Affiliations:** ^1^ Department of Epidemiology and Biostatistics School of Public Health, KCMC University Moshi Tanzania; ^2^ Department of General Surgery School of Medicine, KCMC University Moshi Tanzania; ^3^ Department of Internal Medicine School of Medicine, KCMC University Moshi Tanzania; ^4^ Department of Clinical Physiology School of Medicine, KCMC University Moshi Tanzania; ^5^ Department of Radiology School of Medicine, KCMC University Moshi Tanzania

**Keywords:** imaging, prophylaxis, radiation, radiotherapy, vitamin C

## Abstract

**Background and Aim:**

The introduction of radiation in medicine has led to significant breakthroughs in diagnostic, interventional, and therapeutic practices. However, the increasing demand for medical radiation has also raised concerns regarding cumulative exposure among both patients and healthcare workers, potentially leading to serious long‐term complications, including cancer. This article presents perspectives on the radioprotective potential of ascorbic acid (vitamin C) and discusses its possible role in mitigating the effects of ionizing radiation among at‐risk populations, including patients and healthcare workers.

**Methods:**

Relevant literature addressing the short‐ and long‐term consequences of exposure to ionizing radiation, as well as studies exploring the radioprotective potential of ascorbic acid, was reviewed. Articles aligned with the main objective were synthesized to inform the perspectives presented in this paper.

**Results:**

Several strategies are currently employed to ensure radiation safety for both patients and healthcare workers. However, even when these measures are properly implemented, they do not guarantee complete protection from radiation exposure. Ascorbic acid (vitamin C), a potent antioxidant, has been widely studied for its therapeutic properties, and existing literature suggests that it may play a significant role in protecting against and reducing the harmful effects of ionizing radiation in susceptible individuals.

**Conclusion and Recommendations:**

Vitamin C is a potent antioxidant with demonstrated radioprotective properties that may help reduce the harmful effects of ionizing radiation. Routine supplementation or increased dietary intake of vitamin C may benefit patients and healthcare workers at elevated risk of radiation exposure. However, further clinical trials are needed to determine its prophylactic efficacy, optimal dosage, and intake frequency before it can be incorporated as a standard radioprotective strategy in clinical and other high‐exposure settings.

## 1 Introduction

The discovery of radiation dates back to the end of the nineteenth century and has ever since been consistently used in different fields like medicine [1]. Exposure to radiation can be from natural sources like the sun or artificial as occupational or medical radiological exposure. The exposure to artificial radiation can be due to planned or accidental situations that occur in our daily lives and it has been reported to have increased globally recently [2]. Both diagnostic and therapeutic medical use of radiation is responsible for 98% of the population dose contribution from all artificial sources and accounts for 20% of the total population exposure [3].

The introduction of radiation use in medicine brought a great breakthrough in diagnostic, interventional, and therapeutic advancement [1]. In the diagnostic area, radiations are used in obtaining images for diagnoses and monitoring of different medical conditions [4]. The radiological diagnostic approaches include X‐rays and CT scans. Radiation can be used for therapeutic purposes such as cancer treatment. Radiation therapy contributes up to 40% of curative treatment and it is estimated that 50% of cancer patients will receive radiotherapy during the course of illness [5]. Annually, more than 3600 million diagnostic radiology examinations are performed, 37 million nuclear medicine procedures are carried out, and 7.5 million radiotherapy treatments are given globally [3].

Despite all the merits of radiation in medicine, no amount of radiation exposure is safe for the human body and exposure to this radiation is associated with various short‐ and long‐term risks posed to both patients and Healthcare Workers (HCWs) alike [4, 6, 7]. This raises an alarm of finding protective ways to navigate this threat.

In the medical field where radiation use is of clinical significance and most of the time unavoidable, there are individuals who are more likely to encounter significant radiation exposures [6]. These include patients with certain medical conditions undergoing either diagnostic or therapeutic radiological interventions, and HCWs who regularly encounter various medical procedures involving radiation. Globally, 2.3 million HCWs are working with radiation; thus 50% of all HCWs are exposed to artificial and ionized radiation [8].

Exposure to higher doses of radiation has been associated with some harmful acute side effects such as impairment of tissues and organs mostly manifested as skin inflammation, hair loss, and radiation burns [3, 6]. Even in lower doses, there is still long‐term risk such as skin cancer. HCWs and patients who are frequently exposed to medical radiation are not only at increased risk of cancer development later in life but also genetically predisposing their subsequent generations to cancer [9, 10, 11, 12, 13].

The literature, past and present, has examined the increased risk of cancer among HCWs resulting from occupational exposure to ionizing radiation. Epidemiological data suggest that HCWs exposed to radiation exhibit a significantly higher incidence of cancer compared to nonexposed populations [14, 15, 16, 17, 18, 19, 20]. Notably, a systematic review published in 2020 reported that cancer rates in this group may be as high as 40% [20]. The most frequently reported malignancies include breast cancer (particularly among female workers), brain cancer, melanoma, lymphoma, and leukemia.

Of note, ionizing radiation has been classified as a Group 1 carcinogen (carcinogenic to humans) by the International Agency for Research on Cancer (IARC) and is also recognized by the International Labor Organization (ILO) as one of the principal occupational carcinogens [21, 22].

To mitigate the health risks associated with occupational radiation exposure, the International Commission on Radiological Protection (ICRP) established dose limits for workers beyond which the risk of adverse effects significantly increases. According to the ICRP, the recommended maximum occupational effective dose is 20 mSv/year, averaged over 5 years (i.e., a limit of 100 mSv over 5 years) with no single year exceeding 50 mSv [7, 23]. However, these limits do not represent a definitive threshold between safe and harmful levels of radiation exposure. Instead, exceeding the recommended limits indicates that the probability of health effects (in particular, cancer) surpasses an internationally accepted risk level. Moreover, effective implementation of these guidelines requires that workers consistently wear personal dosimeters to monitor their exposure to ionizing radiation, a practice that is often impractical or inconsistently applied in developing countries.

There are number of ways that have been designed to minimize the exposure to radiation among patients and HCWs, which are in terms of exposure justification, optimization, and dose limitation approaches [6]. They include the use of shielding devices like lead glasses, ceiling‐suspended shields, lead wall installment in radiation rooms, and lead coats dressed by surgeons or radiologists [24, 25]. Also, to minimize radiation exposure to patients, dose optimization has been recommended such as eliminating a patient dose that is higher than necessary which also has an impact on reducing exposure of radiation to medical workers [6, 26, 27]. However, all these approaches provide absolutely no guarantee of complete protection against radiation even when these strategies are implemented or adhered to.

This article aims to highlight the need for effective preventive and control measures against the post‐radiation exposure consequences among patients and HCWs by emphasizing the role of vitamin C supplementation in the prevention and control of radiation‐induced short‐ and long‐term complications before and after radiation exposure.

## 2 Radiation Exposure Consequences

The severity of radiation effects depends on the amount or dose of radiation received [7, 28]. There are two types of responses that have been associated with exposure to ionizing radiation, tissue reaction (deterministic effect) and stochastic effect. Tissue reaction is when the severity of radiation‐induced damage increases proportionately with an increase in radiation dose, and usually occurs when the dose exceeds a particular threshold [7]. Beyond certain thresholds, radiation impairs the functioning of tissues and organs producing acute effects like skin burns, hair loss, hematopoietic damage, or acute radiation syndrome [7, 28, 29]. At higher radiation doses these effects can be even more severe. Exposure to radiation above the tissue/organ tolerance thresholds brings about acute onset complications. On the other hand, stochastic effects, including cancer, are caused by a mutation or other permanent changes usually as a result of prolonged repeated exposure to low doses of radiations [7, 30, 31, 32]. They do not normally depend on radiation dosage threshold, however, the probability of stochastic effect increases with an increase in radiation dosage [7]. These effects are referred to as late‐onset disorder.

Several mechanisms have been proposed to explain how ionizing radiation causes damage to the human body. Radiation can induce either direct or indirect cellular damage. Direct cellular damage occurs when ionizing radiation interacts directly with cellular components, such as DNA or proteins, potentially leading to cell death or genetic mutations. In contrast, indirect damage results from the production of reactive oxygen species (ROS), particularly hydroxyl radicals, generated when radiation interacts with water molecules within the cell. These ROS can subsequently damage cellular structures, including DNA. Notably, approximately two‐thirds of radiation‐induced DNA damage is attributed to these indirect effects mediated by freely diffusing hydroxyl radicals [33].

The initial insult is thought to be secondary to the genome damage such as the radiation‐induced DNA double‐strand breaks (DSBs) [29]. Some studies have proposed that most of these damage results to mutations via DNA deletions [34]. DSBs are the most significant consequence of radiation exposure and the potential mechanism of chromosomal alterations and carcinogenesis [35]. Moreover, it was revealed that our cells can recognize genome damage and induce multiple diverse responses so as to maintain genome integrity [29]. This DNA damage response induces cell cycle arrest, DNA repair, apoptosis, and cell senescence. This response aims to maintain genome integrity but in turn, the death of numerous cells occurs and results in acute disorder. This is followed by DNA miss‐repair and mutation presumed to cause cancer later. In fact, several in vivo studies among humans have clearly demonstrated this effect using γ‐H2AX foci to represent DSB as a biomarker of radiation exposure [35, 36, 37, 38, 39, 40].

DNA DSBs are the most serious radiation‐induced consequence and the potential mechanism of chromosomal alterations and carcinogenesis. The damaged human genetic material leads to both somatic and germline mutations which can be transferred from one generation to another and subsequently leads to cancer and other congenital abnormalities in the future [13]. Most common cancers associated with exposure to the radiations are leukemia, breast, bladder, colon, liver, lung, esophagus, ovarian, multiple myeloma, and stomach cancers [31]. Moreover, prenatal exposures to ionizing radiation may induce brain damage in fetus especially at gestational age between Weeks 8 and 15 [28].

For cancer patients undergoing radiotherapy for curative purposes, it is observed that cancer cells are more radiosensitive to the ionizing radiation. This is the most supporting justifiable explanation for using this mode of treatment. However, for better treatment outcomes, the intensity of rays most of times is increased and subject even the normal cells to the risk of DNA damage on exposure to radiation [28]. Radiotherapy affects rapidly dividing cells like hematopoietic stem cells causing anemia, hematopoietic malignancies, and immunity suppression [34, 41, 42, 43, 44]. Apart from acute radiation side effects faced by these patients, it is undeniable fact that this treatment modality also poses the risk of developing other malignancies such as leukemia.

## 3 Ascorbic Acid and Radiation Exposure

Among other antioxidants, radiation plays a significant role in lowering vitamin C serum levels in the body. A study examining the serial serum levels of vitamins A, E, and C in cancer patients, prior to the initiation of concurrent chemo‐radiotherapy, during treatment, and immediately post‐therapy, reported a significant reduction in the levels of these vitamins throughout the treatment period [45]. Notably, serum levels were already reduced before therapy and declined further during and after treatment. This observation may suggest a potential protective role of vitamin C against radiation‐induced damage during and following exposure. The observed decline in serum vitamin C levels is likely attributed to increased oxidative stress associated with the treatment process.

Despite some measures and strategies in place toward minimizing radiation exposure from interventional radiological procedures in the medical field such as dose reduction, there is still an increase in the demand of using these interventions in the medical field. This is accompanied by an increased risk of facing the consequences of radiation exposure. Radiation exposure leads to radiation‐induced DNA damage secondary to oxidative stress induced by free radicals [35]. On exposure to ionizing radiation, a body produces free radicals and ROS which are the key players in cell damage. This mechanism accounts for about two‐thirds of all radiation‐induced cellular damage [33]. To address these deleterious effects, a significant concentration of antioxidants is required within the body. Vitamin C is widely known as a potent antioxidant and free radical scavenger and it has proven its potential to scavenge these radical products and protect against radiation‐induced cell damage [35, 46, 47]. Moreover, it can even restore the antioxidant properties of other antioxidants like vitamin E [48]. This effect has been repeatedly demonstrated in animal models showing decreased complications and mortality among vitamin C‐treated groups prior and post‐radiation exposure [46, 47, 49, 50]. In these experimental studies, not only prevention, but treatment of radiation‐induced DNA damage has been demonstrated. Vitamin C has even showed radioprotective effect similar to that of glutathione which is the most important antioxidants in human body [51].

In humans, a number of experimental studies have found that radiation‐induced DNA damage can be prevented as well as reversed by vitamin C as an antioxidant. A study by Brand and colleagues demonstrated the influence of antioxidants on X‐ray‐induced DNA DSBs using γ‐H2AX foci as a biomarker of radiation‐induced damage [35]. Vitamin C administered prior to irradiation demonstrated potential to reduce γ‐H2AX foci in blood lymphocytes. Another study also concluded that administration of oral vitamin C to the patients who have underwent abdominal contrast enhanced CT examination significantly reduces the level of DSB and is a simple and effective method to decrease DNA damage [40]. In addition to that, a review which aimed to look on the radioprotective effect ex vivo, in vitro, and in vivo of vitamins concluded that vitamin C among other vitamins (A, D, and E) in its natural form or supplementation can be useful to reduce the radiation effect in the body, organs, and/or cells [51].

## 4 Discussion

With the increase in radiation use in the medical field, the consequences secondary to radiation exposure will also increase [3]. It is evident that the available strategies to prevent the population from radiation exposure are not effective enough to halt these consequences [7]. Moreover, the most serious and feared consequence which is cancer can develop even at very low levels post‐radiation exposure after several years [13]. Physicians, patients, orthopedic surgeons, and radiologists represent the most vulnerable population. It is even worse that the radiation effects like cancer development can also affect future generations of exposed individuals even at low dose radiation exposure [4, 7, 12, 13, 30]. These findings highlight the need for a more efficient and ideal solution amidst this low‐profile problem.

Several studies have demonstrated and therapeutically suggest that Vitamin C administration both pre‐ and post‐exposure to either diagnostic or therapeutic radiation is clinically useful significantly in preventing and controlling both long‐ and short‐term post‐radiation exposure consequences [40, 46, 47, 49, 50]. Such findings imply clinically that vitamin C can be supplemented routinely as prophylaxis to HCWs like radiologists who are routinely exposed to radiation and patients who have regular radiotherapy schedules for prevention and control of both short‐ and long‐term post‐radiation exposure sequelae.

## 5 Conclusion and Recommendation

There is a growing demand for ionizing radiation in medical practice, increasing the risk of serious complications such as DNA damage, genetic mutations, impaired immunity, and cancer in both patients and HCWs. Therefore, unnecessary radiation exposure should be avoided, especially when alternative diagnostic or therapeutic options are available. Strict adherence to radiation safety principles including justification, dose optimization, and dose limitation is essential to protect patients and medical professionals.

Vitamin C is a potent antioxidant with demonstrated radioprotective effects, helping to reduce the harmful consequences of ionizing radiation. Supplementation before and around the time of radiation exposure may minimize short‐ and long‐term radiation‐induced complications. Additionally, HCWs at high risk of occupational exposure such as radiologists, physicians, and orthopedic surgeons may benefit from regular vitamin C supplementation or routine consumption of vitamin C‐rich foods.

Beyond existing radiation safety measures, clinical trials are needed to evaluate more the prophylactic role of ascorbic acid, including optimal dosage, timing, and intake frequency. Research should also determine whether vitamin C is most effective when administered before, after, or both before and after radiation exposure, as well as establish appropriate supplementation schedules for HCWs at high occupational risk.

## Author Contributions

Harold L. Mashauri conceptualized the manuscript, administered and supervised the project. All authors did data curation. Justice Mushemba, Jaynes F. Kabuhaya, and Cornel M. Angolile wrote the first draft of the manuscript. Harold L. Mashauri reviewed and corrected the first draft. All authors have read and approved the final version of the manuscript.

## Funding

The authors received no specific funding for this work.

## Disclosure

The lead author Cornel M. Angolile affirms that this manuscript is an honest, accurate, and transparent account of the study being reported; that no important aspects of the study have been omitted; and that any discrepancies from the study as planned (and, if relevant, registered) have been explained.

## Conflicts of Interest

The authors declare no conflicts of interest.

## Data Availability

The authors have nothing to report.
